# The effect of major depressive disorder comorbidity on ischemia-modified albumin levels, a marker of oxidative stress, and antioxidant defense system in patients with obsessive compulsive disorder

**DOI:** 10.3389/fpsyt.2024.1469640

**Published:** 2024-10-15

**Authors:** Sertaç Zengil, Esra Laloğlu

**Affiliations:** ^1^ Department of Psychiatry, University of Health Sciences Erzurum Faculty of Medicine, Erzurum, Türkiye; ^2^ Department of Medical Biochemistry, Ataturk University Faculty of Medicine, Erzurum, Türkiye

**Keywords:** antioxidant enzymes, ischemia-modified albumin, major depressive disorder, malondialdehyde, obsessive-compulsive disorder, oxidative stress

## Abstract

**Objective:**

The aim of this study was to examine the levels of oxidant and antioxidant markers in patients with obsessive-compulsive disorder (OCD) and to investigate whether these levels change in the presence of major depressive disorder (MDD) comorbidity.

**Methods:**

This study was completed with 23 OCD patients with MDD comorbidity (OCD+MDD), 21 OCD patients without MDD comorbidity (OCD-MDD) and 21 healthy controls. Oxidative stress levels of the cases’ were determined by ischemia modified albumin (IMA) and malondialdehyde (MDA) measurements and antioxidant levels were determined by superoxide dismutase (SOD), catalase (CAT) and glutathione peroxidase (GSH-Px) measurements. One-way analysis of variance (ANOVA) and unpaired Student’s t-test were used to compare the study groups. *Post hoc* Bonferroni test was used for the degree of significance between groups, and repeated measures analysis of covariance (ANCOVA) was used to investigate the effect of age and gender.

**Results:**

IMA and MDA levels were significantly higher in the OCD group compared to the control group, and SOD, CAT and GSH-Px levels were lower in the OCD group compared to the control group (p<0.01). IMA levels were significantly higher in the OCD+MDD group compared to the OCD-MDD group, while SOD, CAT and GSH-Px levels were significantly lower in the OCD+MDD group compared to the OCD-MDD group (p<0101). MDA levels were significantly higher in the OCD+MDD group compared to the OCD-MDD group (p=0.009). When the entire OCD patient group was examined, significant, powerful, positive correlations were observed between Y-BOCS and HDRS scores and IMA and MDA, and significant powerful negative correlations between Y-BOCS and HDRS scores and SOD, CAT, and GSH-Px (p<0.001 for all). In OCD+MDD group, oxidative stress markers increased significantly in parallel with the severity of depression, while antioxidant levels decreased (p=0.003 for IMA, p<0.001 for others).

**Conclusions:**

We believe that parameters indicating impaired oxidant/antioxidant balance in patients with obsessive-compulsive disorder may help to elucidate the cause of the disease and may be potentially useful biomarkers in the diagnosis and determination of the severity of comorbid MDD.

## 1 Introduction

Obsessive-compulsive disorder (OCD) is a disorder characterized by involuntary, disturbing and repetitive thoughts (obsessions) that are foreign to the self and cannot be removed from the mind with conscious effort and involuntary actions taken to get rid of the disturbing effects of these thoughts. The lifelong prevalence of OCD is approximately 2-3% (1). OCD is an important condition due to its being frequently encountered, progressing with functional impairment in all areas, and resulting in severe disability.

The condition is frequently accompanied by other mental disorders, particularly major depressive disorder (MDD) (2). The etiology of OCD is multifaceted and includes genetic, biological and psychosocial factors. Functional impairments in structures such as the orbitofrontal cortex of the brain, the limbic system, basal ganglia, and thalamus are thought to occur in the etiology of OCD, and neurotransmitters such as the dopamine and serotonin system and various neuropeptides have been implicated in these disorders (3). Although antidepressants with anti-obsession effects (especially serotonin reuptake inhibitors) and cognitive-behavioral therapy have been shown to be highly effective in the treatment of OCD, the inadequate treatment response of a significant proportion of patients, including approximately 40-60% of patients, increases the interest in studies related to the neurobiology of OCD (4). The neurobiology needs to be better understood in order to overcome difficulties in diagnosis. Recent accumulating evidence shows that oxidative stress exhibits an effect in numerous psychiatric diseases, including OCD (5). It is known that brain tissue is much more sensitive to oxidation than other tissues. The high unsaturated fatty acid content in the brain, the formation of free radical by-products as a result of high oxygen use, high lipid peroxidation and weak antioxidant defense system, the high reducing potential of various neurotransmitters, and the presence of redox-catalytic metals such as iron and copper can be counted among the reasons why the brain is more sensitive to oxidation. Oxidative damage occurring in the brain can alter the functions of neurons and make individuals more prone to both neurodegenerative and neuropsychiatric diseases (6).

Free radicals form during normal cell metabolism in the human body. A balance, known as oxidative stability, exists between the rates of free radical formation and elimination. Oxidative stress is defined as the deterioration of the oxidative balance due to the increase in reactive oxygen species such as hydroxyl radical, superoxide radical and hydrogen peroxide formed during cellular metabolism and the inadequacy of antioxidants that detoxify them (7). Various trace elements such as antioxidant vitamins E, C and A, glutathione (GSH), selenium, metalloenzyme-containing glutathione peroxidase (GSH-Px), iron-containing catalase (CAT) and superoxide dismutase (SOD) play a protective role against free radical-induced cell damage (8). In another study, it was found that there was an imbalance in antioxidant enzymes in bipolar depression, CAT and GPx increased, and the SOD/CAT ratio decreased, and a reactive increase in antioxidant enzyme levels was shown in the same patient group with the treatment of bipolar disorder (9). In addition, there are studies that provide strong evidence for the role of inflammation in the pathophysiology of acute mania in bipolar disorder (10). Increasing evidence highlights the relationship between inflammation and suicidal behavior, showing that oxidative stress parameters increase with the severity of suicidal behavior (11). Similarly, there are also studies supporting the fact that oxidative stress is significantly higher in schizophrenia patients, especially those in acute attacks, compared to stable patients and that this situation contributes to the pathophysiology of schizophrenia (12).

Malondialdehyde (MDA) is a degradation product of large chain reactions that lead to the oxidation of polyunsaturated fatty acids. With this feature, MDA is one of the most reliable markers of oxidative stress (13). Superoxide dismutase, CAT, and GSH-Px have been measured as antioxidants and MDA as an oxidative stress marker in numerous studies of psychiatric diseases (14, 15).

Ischemia modified albumin (IMA) is calculated by binding cobalt to albumin. While IMA was previously considered a biomarker of ischemia caused only by tissue hypoxia, recent studies have shown that IMA is a reliable indicator of not only ischemia but also oxidative stress (16–18). In recent years, there have been a limited number of studies evaluating IMA levels in psychiatric diseases. A recent study with bipolar disorder patients reported that IMA levels were significantly higher in acute mania and early remission compared to healthy controls (19). In addition, it was reported that IMA levels were increased in both schizophrenia and bipolar disorder patients, these higher levels were statistically significant only in bipolar disorder patients, and IMA elevation in schizophrenia and bipolar patients may be related to metabolic parameters (20). In a recent study conducted in the pediatric age group, it was reported that no significant difference was observed in thiol-disulfide homeostasis and IMA levels between the MDD and control groups (21). However, in the literature search, no other study investigating IMA levels in the MDD patient group was found other than this study, and there was also no study investigating IMA levels in OCD patients.

The findings obtained to date confirm the link between oxidative stress and OCD (14, 15). However, further research is needed to clarify this link in relation to various aspects of psychopathology. In this study, which we planned with the hypothesis that oxidative stress parameters would be higher in OCD patients, we aimed to examine the levels of antioxidants CAT, SOD and total GSH and oxidative stress markers MDA and IMA in OCD patients and to investigate whether there is a relationship between oxidative parameters. In addition, since the known effect of oxidative stress in MDD pathophysiology and the frequent MDD comorbidity in OCD may be a confounding factor in our data, we aimed to compare OCD patients with and without MDD comorbidity within themselves and with healthy controls in this study.

## 2 Methods

This study was approved by the Ethics Committee of Atatürk University Faculty of Medicine (no. B.30.2.ATA.0.01.00/375 date and 30.03.2023). The study was conducted at the Health Sciences University Erzurum Medical Faculty Mental Health and Diseases Department and the Ataturk University Medical Faculty Department of Biochemistry. The study was conducted in accordance with the principles of the Declaration of Helsinki. Informed consent was obtained from all patients and controls before inclusion in the study.

### 2.1 Sample size

In this study, the sample size was calculated using G Power 3.1.9.7 (22). With an effect size of 0.8, a power of 80%, and an alpha error of 0.05, the required sample size for this study was calculated to be 21 per group. The present study included minimum of 21 subjects in each group.

### 2.2 Patients

Forty four patients presenting to the Health Sciences University Erzurum Medical Faculty Mental Health and Diseases Clinic and diagnosed with OCD on the basis of the Diagnostic and Statistical Manual of Mental Disorders, Fifth Edition (DSM-5) were included in the study. The Structured Clinical Interview for DSM-5 Disorders—Clinician Version (SCID-5/CV) was used to investigate psychiatric disorders based on DSM-5. The patients were OCD patients between the ages of 18-65 and had no psychiatric comorbidities other than MDD. None of the patients had used any medication or received any other treatment in the last month. The patients were screened for the presence of MDD on the basis of DSM-5 and were accordingly divided into two groups – OCD patient group with MDD comorbidity (OCD+MDD), OCD patient group without MDD comorbidity (OCD-MDD). This study was completed with 23 OCD+MDD and 21 OCD-MDD patients. Those with an additional psychiatric comorbidity other than MDD, those with any cardiac disease and any ischemic disease that may affect IMA level, those with chronic disease or neurological disease that may affect oxidative stress parameters, those with limited mental capacity and pregnant women were not included in the study because they may confound the study results.

### 2.3 The controls

The controls consisted of 21 individuals who applied to the Health Sciences University Erzurum Medical Faculty Psychiatry Clinic and were evaluated as mentally and physically healthy. Participants were evaluated by a psychiatrist. SCID-5/CV was used to exclude psychiatric disorders based on DSM-5. The healthy controls included in the study were matched with the patient group in terms of age and gender. Those who used alcohol or cigarettes, used any antioxidant agent, and had chronic systemic or neurological diseases were not included in the study because they could have confounding effects on the results. In addition, individuals with serum albumin levels below 2 g/dl and above 5.5 g/dl were excluded from the study.

### 2.4 Data collection tools

#### 2.4.1 The Structured Clinical Interview for DSM-5 Disorders—Clinician Version

It is a clinician-administered, semi-structured clinical interview guide developed for DSM-5 based diagnoses of psychiatric disorders. This guide was developed by First et al., and its Turkish language validity and reliability were performed by Elbir et al. (23, 24).

#### 2.4.2 Sociodemographic data form

This form was prepared by us in line with the literature scanned and the clinical information obtained. It was used to investigate the clinical and sociodemographic characteristics of the participants. It is a semi-structured form that includes sociodemographic information such as gender, age, marital status, as well as clinical data such as age of onset of the disease, duration of the disease, number of hospitalizations during the disease, and treatments received.

#### 2.4.3 Yale-Brown Obsessive-Compulsive Scale

This scale was developed by Goodman and colleagues to measure the type and severity of symptoms in OCD patients (25, 26). Its Turkish validity and reliability were conducted by Karamustafalıoğlu and colleagues (27). It is a semi-structured scale administered by the clinician. In our study, Y-BOCS consisting of 10 items was used. Items 1-5 of these items assess obsessions, and items 6-10 assess compulsions. Each item is scored out of four points, and a maximum of 40 points can be obtained as a total score. YOBCS score cut-off points were determined as 0–7 non-clinical level; 8–15 mild; 16–23 moderate; 24–31 severe; 32–40 extremely severe. The intraclass correlation coefficients of each scale are 0.88. Cronbach Alpha reliability coefficients for the sub-dimensions were found to be 0.96, 0.94, 0.93, 0.95, 0.95, and 0.94, respectively. It was observed that the discriminant validity of the scale was sufficient and it showed a high correlation with the sub-scales.

#### 2.4.4 Hamilton Depression Rating Scale

This scale was used to measure the severity of depressive symptoms. The scale was developed by Hamilton (28). Its Turkish validity and reliability were made by Akdemir et al. (29). It is a semi-structured scale administered by the clinician. It consists of 17 questions and each question is scored between 0-4. The maximum score that can be obtained from the scale is 51. HDRS score cut-off points were determined as 0-7 points as no depression, 8-15 points as mild depression, 16-28 points as moderate depression, and 29 and above as severe depression. The Cronbach alpha internal consistency coefficient of the scale was found to be 0.75, and the reliability coefficient according to the Spearman-Brown formula was found to be 0.76 (29).

### 2.5 Measurement of biochemical and oxidative stress parameters

Blood samples of the patient and control groups were taken between 07:00 and 09:00 in the morning after a 12-hour fast. Venous blood from the antecubital fossa of all patients and controls was collected into ethylenediamine tetraacetic acid (EDTA) tubes.

#### 2.5.1 Preparation of plasma and washed erythrocyte hemolysate

Blood specimens placed into (EDTA) tubes were centrifuged for 10 min at 5000 rpm. Serum and erythrocytes were separated. The plasma and leukocyte layers in the upper part of the tubes were removed using a pipette. The resulting erythrocytes were hemolyzed with five times their volume of ice water. This was followed by centrifugation for 30 min at +4°C at 15,000 rpm to remove the cell membranes. The supernatant was removed using a pipette. The hemolysate was thus prepared. All these procedures were carried out at +4°C. The erythrocyte hemolysates obtained were then stored at -80°C until being used to determine GSH-Px, CAT, and SOD levels, while the plasma was used to measure MDA. Blood placed into sterile gel tubes was centrifuged for 10 min at 4000 rpm, and the resulting sera were stored at -80°C for IMA measurement.

#### 2.5.2 Hemolysate hemoglobin assay

The ferricyanide in Drabkin solution oxidizes the +2-valent iron in the hemoglobin to +3 and converts the hemoglobin to methemoglobin. Methemoglobin combines with potassium cyanide to yield the stable molecule cyanomethemoglobin. The absorbance of cyanomethemoglobin measured at 540 nm is directly proportional to the amount of hemoglobin present (30).

#### 2.5.3 Determination of plasma MDA levels

Plasma MDA levels were measured using the method described by Ohkawa et al. (31). This relies on the principle of measuring the absorbance of the color formed by MDA with thiobarbituric acid in an acidic medium at a wavelength of 532 nm. The MDA results were expressed as nmol/L.

#### 2.5.4 Measurement of GSH-Px activity

GSH-Px activity levels in hemolysates of erythrocytes were measured using Paglia and Valentine’s method (32). The oxidation of nicotinamide adenine dinucleotide phosphate was determined spectrophotometrically at 340 nm at 37°C. Results for specific activity were divided by g/Hb for erythrocyte specimens. The results were expressed as U g^–1^ Hb.

#### 2.5.5 Measurement of CAT activity

Aebi’s method was applied, in which CAT activity is determined using a spectrophotometric method based on the breakdown of hydrogen peroxide by catalase (33). Results for specific activity were divided by g/Hb for erythrocyte specimens. The activities were expressed as kg^−1^ Hb.

#### 2.5.6 Measurement of IMA Levels

Serum IMA levels were measured spectrophotometrically based on the principle of albumin’s cobalt-binding capacity, as described by Bar-Or et al. (34). The results were expressed as absorbance units (ABSUs). In this method, since the rate of unbound cobalt will decrease in patients with high albumin, IMA is measured as low. Therefore, in the literature, albumin-corrected IMA index (Albumin X 23 + IMA - 100) is calculated or patients with serum albumin levels below 2 g/dl and above 5.5 g/dl are excluded to prevent interpersonal albumin level changes from affecting the IMA amount. In our study, patients with serum albumin levels below 2 g/dl and above 5.5 g/dl will be excluded so that IMA levels are not affected by low and high albumin levels.

#### 2.5.7 Determination of SOD activity

Erythrocyte hemolysates were used for the measurement of total (Cu-Zn and Mn) SOD activity levels, as described by Sun et al. (35). This method is based on the reduction of superoxide, which is produced by the xanthine oxidase enzyme system, with nitroblue tetrazolium (NBT). One unit of SOD was determined as the amount that causes a 50% decrease in NBT. Results for specific activity were divided by g/Hb for erythrocyte specimens. The results were expressed as U g^–1^ Hb.

### 2.6 Statistical analysis

SPSS 20.0 for Windows software (SPSS Inc.) was employed for data recording and statistical analysis. Descriptive statistics were expressed as number and percentage for categorical variables and as mean(SD) for numerical variables. Normality of distribution was evaluated based on visual (histograms, probability plots) and analytical methods (Kolmogorov-Smirnov/Shapiro–Wilk tests. One‐way analysis of variance (ANOVA) and the unpaired Student’s t-test were used to compare the study groups. The degree of significance between groups was determined using the *post hoc* Bonferroni test. Also repeated measures analysis of covariance (ANCOVA) were used to investigate the effect of age and sex as covariates. Correlation analyses were performed using Pearson’s correlation test. p values < 0.05 were regarded as statistically significant.

## 3 Results

Forty-four patients (26 women and 18 men) and 21 healthy controls (12 women and nine men) were included in the study. The patients’ and controls’ sociodemographic and clinical characteristics are shown in **Table 1**. No significant differences in terms of age or sex were observed between the groups (p=0.74 and p=0.28, respectively).

**Table 1:** A comparison of the groups’ sociodemographic and clinical characteristics.

	*N*	*%*	*mean (SD)*
Total patients	44		
Gender male	18	40.9%	
female	26	59.1%	
Age, years			31.26 (8.56)
Duration of illness, years			5.31 (5.11)
Subgroups			
OCD + MDD	23	52.3%	
OCD – MDD	21	47.7%	
Scale scores			
HDRS score	23		29.00 (4.64)
Y-BOCS score	44		23.77 (8.84)
OCD + MDD group according to HDRS			
Moderate depression	10	43.5%	
Severe depression	13	56.5%	
According to Y-BOCS			
Mild-moderate OCD	19	43.2%	
Severe OCD	15	34.1%	
Significant severe OCD	10	22.7%	
Control group			
Gender male	9	42.9%	
female	12	57.1%	
Age, years	21		33.90 (9.43)

Data are expressed as numbers and percentages (%).

OCD, Obsessive-compulsive disorder; MDD, Major depressive disorder; OCD+MDD, (OCD patient group with MDD comorbidity); OCD-MDD, (OCD patient group without MDD comorbidity); HDRS, Hamilton Depression Rating Scale; Y-BOCS, Yale-Brown Obsessive-Compulsive Scale.

**Figure 1:**
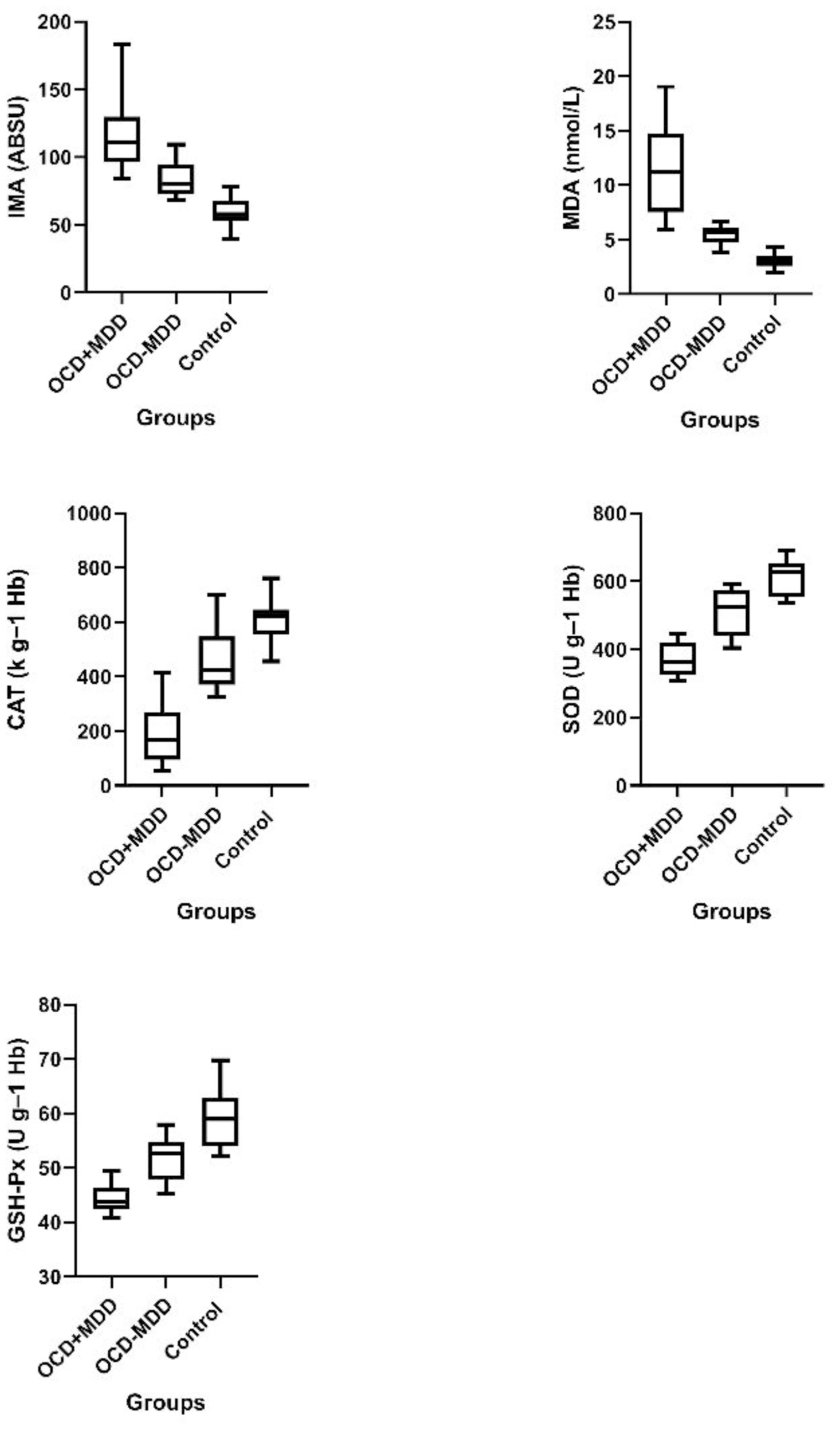
Box-plot representation of oxidant and antioxidant levels in OCD+MDD, OCD-MDD and control groups. IMA, Ischemia-modified albumin; MDA, Malondialdehyde; SOD, Superoxide dismutase; CAT, Catalase; GSH-Px, Glutathione peroxidase; OCD+MDD; OCD patient group with MDD comorbidity; OCD-MDD, OCD patient group without MDD comorbidity.

The patients with OCD were divided into two subgroups depending on the presence or absence of MDD comorbidity, OCD+MDD and OCD-MDD. The groups’ IMA, MDA, SOD, CAT, and GSH-Px levels are shown in **Table 2**. MDA and IMA levels were significantly higher in both the OCD subgroups than in the control group, while SOD, CAT, and GSH-Px were lower. IMA and MDA levels were also significantly higher in the OCD patients with MDD comorbidity (OCD+MDD) than in those with no such comorbidity (OCD-MDD), while SOD, CAT, and GSH-Px levels were significantly lower (**Figure 1**). Also in an ANCOVA taking baseline age and sex as covariates, the difference between groups remained significant (all p<0.001).

**Table 2:** OCD+MDD, OCD-MDD and control groups’ IMA, MDA, SOD, CAT, and GSH-Px levels.

*Groups*	*IMA (ABSU)*	*MDA (nmol/L)*	*SOD (U g–1 Hb)*	*CAT (k g−1 Hb)*	*GSH-Px (U g–1 Hb)*
OCD+MDD (n=23)	117.47 (25.37)	11.54 (4.25)	370.94 (48.11)	184.33 (102.05)	44.43 (2.58)
OCD-MDD (n=21)	84.03 (12.24)	5.36 (0.82)	507.33 (67.33)	461.71 (105.57)	51.74 (4.00)
Control group (n=21)	58.72 (9.60)	3.07 (0.68)	604.71 (75.18)	417.90 (107.65)	59.22 (5.48)
Statistics					
1 vs. 2 vs. 3^a^	**p<0.001**	**p<0.001**	**p<0.001**	**p<0.001**	**p<0.001**
1 vs. 2^a^	**p<0.001**	**p<0.001**	**p<0.001**	**p<0.001**	**p<0.001**
1 vs. 3^a^	**p<0.001**	**p<0.001**	**p<0.001**	**p<0.001**	**p<0.001**
2 vs. 3^a^	**P<0.001**	**p=0.009**	**p<0.001**	**p<0.001**	**p<0.001**

1:OCD+MDD group (OCD patient group with MDD comorbidity), 2: OCD-MDD group (OCD patient group without MDD comorbidity), 3: Control group.

^a^ANOVA with Bonferroni HSD. Values expressed as mean (SD).

IMA, Ischemia-modified albumin; MDA, Malondialdehyde; SOD, Superoxide dismutase; CAT, Catalase; GSH-Px, Glutathione peroxidase; ABSU, absorbance unit. Statistically significant p values ​​are indicated in bold.

The OCD patient group was also divided into three subgroups based on Y-BOCS scores – mild-moderate, severe, and very severe. Interestingly, IMA and MDA rose significantly in line with the severity of OCD, while SOD, CAT, and GSH-Px decreased (**Table 3**).

**Table 3:** IMA, MDA, SOD, CAT and GSH-Px levels of groups classified as mild-moderate, severe and very severe according to Y-BOCS scores.

*Groups*	*IMA (ABSU)*	*MDA nmol/L*	*SOD U g–1 Hb*	*CAT k g−1 Hb*	*GSH-Px U g–1 Hb*
Mild-moderate (n=19)	77.82 (6.79)	5.39 (0.91)	534.38 (51.02)	496.50 (97.63)	53.43 (2.89)
Severe (n=15)	101.17 (6.91)	7.32 (2.13)	411.67 (28.48)	286.80 (79.18)	46.25 (1.66)
Very severe (n=10)	139.55 (20.78)	15.55 (2.54)	325.75 (15.55)	96.00 (28.86)	42.19 (0.90)
Statistics					
1 vs. 2 vs. 3^a^	**p<0.001**	**p<0.001**	**p<0.001**	**p<0.001**	**p<0.001**
1 vs. 2^a^	**p<0.001**	**p=0.018**	**p<0.001**	**p<0.001**	**p<0.001**
1 vs. 3^a^	**p<0.001**	**p<0.001**	**p<0.001**	**p<0.001**	**p<0.001**
2 vs. 3^a^	**p<0.001**	**p<0.001**	**p<0.001**	**p<0.001**	**p<0.001**

1:OCD+MDD group (OCD patient group with MDD comorbidity), 2: OCD-MDD group (OCD patient group without MDD comorbidity), 3: Control group.

^a^ANOVA with Bonferroni HSD. Values expressed as mean (SD).

Y-BOCS, Yale-Brown Obsessive-Compulsive Scale; IMA, Ischemia-modified albumin; MDA, Malondialdehyde; SOD, Superoxide dismutase; CAT, Catalase; GSH-Px, Glutathione peroxidase; ABSU, absorbance unit. Statistically significant p values ​​are indicated in bold.

The OCD+MDD group was further subdivided into moderate and severe subgroups based on HDRS scores. Oxidative stress markers increased significantly in line with the disease severity while antioxidant marker levels decreased (**Table 4**).

**Table 4:** IMA, MDA, SOD, CAT and GSH-Px levels in the OCD+MDD group, which was classified as moderate and severe according to HDRS scores.

Groups	IMA (ABSU)	MDA nmol/L	SOD U g–1 Hb	CAT k g−1 Hb	GSH-Px U g–1 Hb
Moderate (n=10)	91.22 (5.53)	6.93 (0.72)	431.8 (10.33)	318.6 (62.29)	47.85 (1.22)
Severe (n=13)	127.56 (22.46)	13.31 (3.63)	347.54 (33.27)	132.69 (55.31)	43.11 (1.47)
Statistics					
1 vs. 2^a^	**p=0.003**	**p<0.001**	**p<0.001**	**p<0.001**	**p<0.001**

^a^Student’s t-test. Values expressed as mean(SD).

1:OCD+MDD group (OCD patient group with MDD comorbidity), 2: OCD-MDD group (OCD patient group without MDD comorbidity), 3: Control group.

HDRS, Hamilton depression rating scale; IMA, Ischemia-modified albumin; MDA, Malondialdehyde; SOD, Superoxide dismutase; CAT, Catalase; GSH-Px, Glutathione peroxidase; ABSU, absorbance unit. Statistically significant p values ​​are indicated in bold.

Duration of OCD was positively correlated with IMA and MDA and negatively correlated with SOD, CAT, and GSH-Px (r=0.484, r=0.523, r= -0.431, r= -0.472, and r= -0.429 respectively, p<0.001 for all). The marker most correlated with disease duration was MDA.

**Figure 2:**
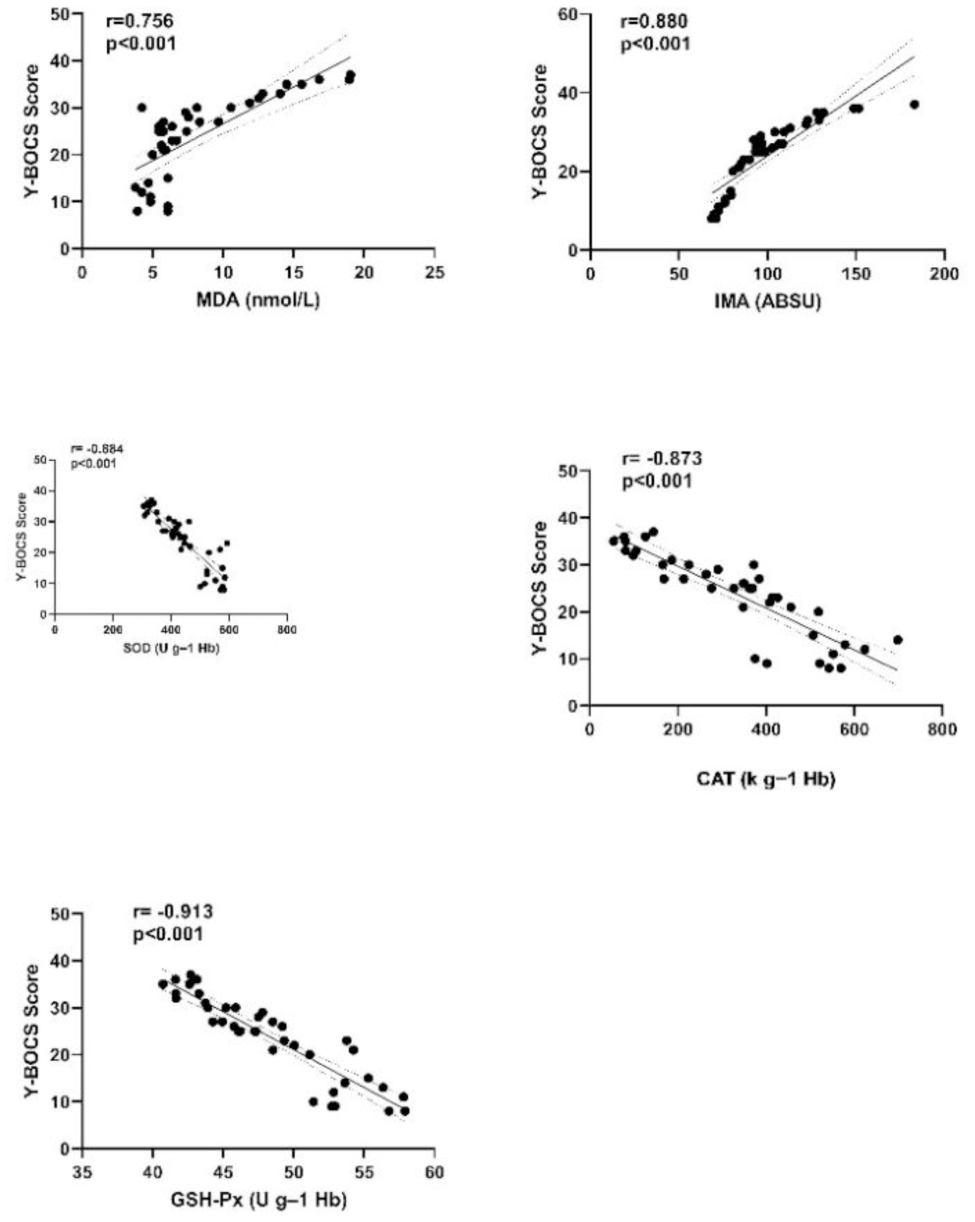
Correlation graph between Y-BOCS scores of OCD patient group and oxidant and antioxidant parameters. Y-BOCS, Yale-Brown Obsessive-Compulsive Scale; IMA, Ischemia-modified albumin; MDA, Malondialdehyde; SOD, Superoxide dismutase; CAT, Catalase; GSH-Px, Glutathione peroxidase.

**Figure 3:**
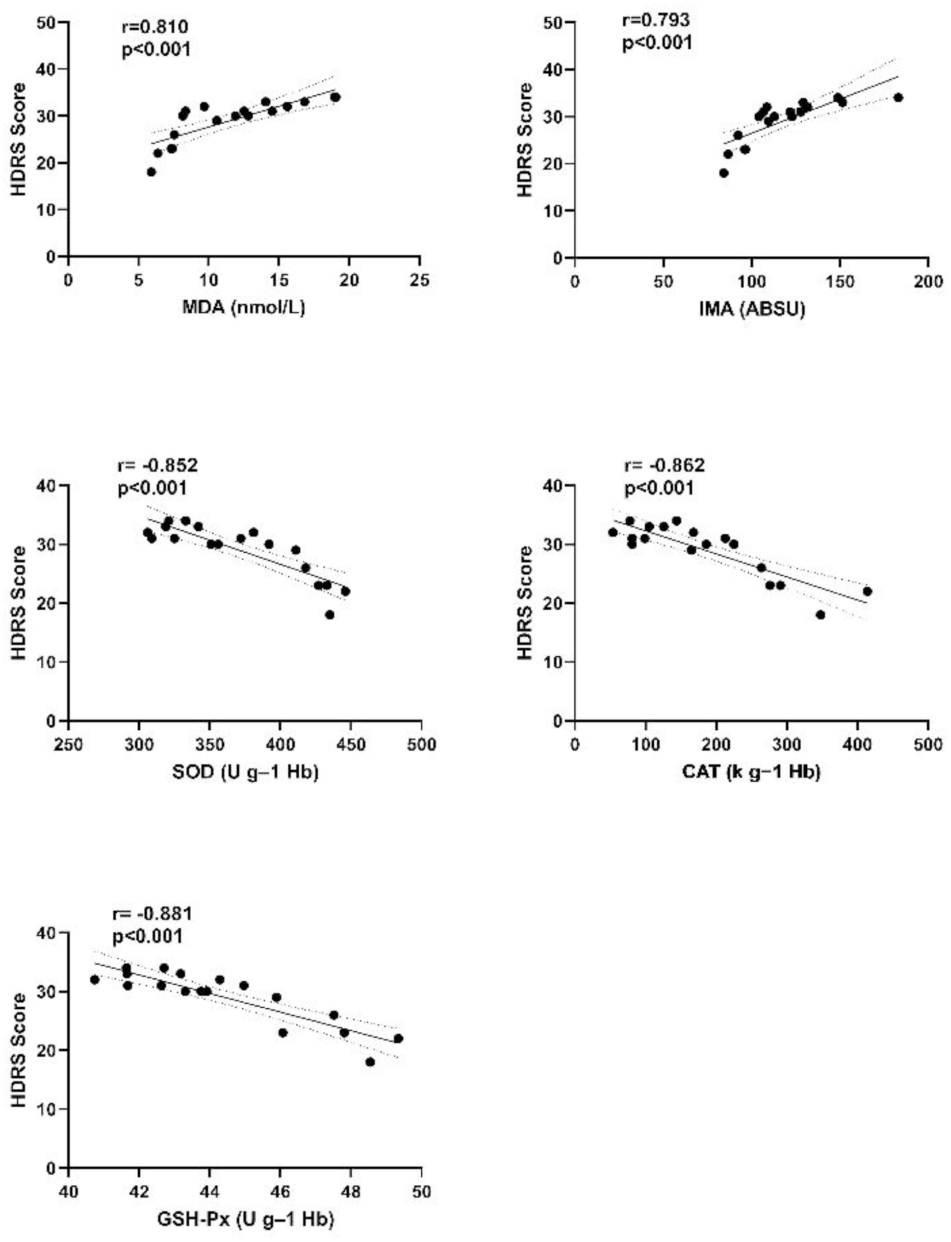
Correlation graph between HDRS scores and oxidant and antioxidant parameters of the OCD+MDD patient group. HDRS, Hamilton depression rating scale; IMA, Ischemia-modified albumin; MDA, Malondialdehyde; SOD, Superoxide dismutase; CAT, Catalase; GSH-Px, Glutathione peroxidase; OCD+MDD; OCD patient group with MDD comorbidity.

When the entire OCD patient group was examined, significant, powerful, positive correlations were observed between Y-BOCS and HDRS scores and IMA and MDA, and significant powerful negative correlations between Y-BOCS and HDRS scores and SOD, CAT, and GSH-Px (r=0.880, r=0.756, r=-0.884, r=-0.873, and r=-0.913, respectively, for Y-BOCS scores, p <0.001 for all; r=0.793, r=0.810, r=-0.852, r=-0.862, and r=-0.881, respectively, for HDRS scores, p <0.001 for all) (**Figures 2**, **3**).

## 4 Discussion

In this study, antioxidants CAT, SOD and GSH-Px levels were found to be significantly lower in the OCD patient group compared to the healthy control group, while oxidative stress markers IMA and MDA levels were found to be significantly higher. This supports the idea of an etiopathogenic effect of oxidative stress in OCD.

A product of lipid peroxidation, MDA is a known biomarker of oxidative stress. Evidence also exists that MDA levels follow a high course in OCD (15). Shohag et al. reported higher levels of MDA and lower levels of vitamins E and C, which exhibit antioxidant effects, in patients with OCD compared to healthy controls (36). This is consistent with the present study.

After innovative studies in recent years, IMA has been identified as a biological marker of ischemia as well as oxidative stress (37). It has been recommended as an oxidative stress-related biomarker in numerous diseases, including myocardial infarction and cerebrovascular diseases, diabetes kidney failure, hypo- and hyperthyroidism, multiple sclerosis, various dermatological diseases, and preeclampsia (38–40). However, when the literature was examined, no previous study investigating serum IMA levels in psychiatric diseases including OCD was found. The present study is therefore particularly important to the literature.

Many conflicting results have been reported in the literature regarding oxidative stress and antioxidant levels in OCD. For example, Selek et al. reported higher antioxidant levels in OCD compared to healthy controls in their study and suggested that this may be due to a rebound phenomenon or the chronicity of the disease (41). There are also studies showing a positive correlation between disease severity and oxidative markers in OCD. For example, one study observed higher levels of nitric oxide, a reactive nitrogen species, and thiobarbituric acid, a product of lipid peroxidation, in patients with OCD, and emphasized that these were related to the severity of the disease (42). Similar to these studies, in our study, it was observed that oxidative stress marker levels increased significantly depending on the severity of the disease, while antioxidant marker levels decreased with the severity of the disease. These data can be interpreted as there is a positive relationship between disease severity and oxidative stress severity in OCD.

Studies evaluating levels of the lipid peroxidation products and antioxidant enzymes CAT, SOD, and GSH-Px in psychiatric diseases have reported very different findings. A study of patients with schizophrenia reported significantly lower CAT and GSH-Px levels compared to healthy controls (43). Another study, however, observed no significant difference between GSH levels in the posterior medial frontal cortex of patients with schizophrenia and those of healthy controls (44). Data have been reported for lower SOD and CAT levels in patients with bipolar disorder than in a healthy control group (45). There are also data in the literature regarding an increase in CAT activity and a decrease in GSH-Px levels in MDD (46). However, limited data have associated SOD, GSH-Px, and CAT activity levels with OCD (47).

Oxidative stress plays a role in the etiology of many neuropsychiatric diseases as well as OCD and is considered an etiopathogenic cause of MDD (46). An inflammatory process is implicated in the pathophysiology of MDD. This inflammatory process includes microglial activation, increased cytokine release, and increased oxidative stress, as well as changes in glutamatergic system regulation that may exacerbate astrocyte atrophy and local damage following these events (48). Studies have found a decrease in antioxidant levels and an increase in oxidative damage products in patients with MDD, and have shown a positive correlation with the severity of the disease and the increased number of depressive episodes. For example, a recent study found higher levels of MDA in MDD compared to healthy controls (49). In our study, IMA and MDA levels were significantly higher in OCD patients with MDD comorbidity, while SOD, CAT and GSH-Px levels were significantly lower than in those without MDD comorbidity. These data show that MDD comorbidity accompanying OCD further raises oxidative stress levels that are already present in OCD. In addition, antioxidant enzyme levels decrease while oxidative stress biomarkers increase as the severity of both OCD and MDD worsens. This indicates the importance of detailed investigation and treatment of comorbid MDD in the treatment of OCD.

In this study, the positive correlation between OCD disease duration and IMA and MDA, and the negative correlation between OCD disease duration and SOD, CAT and GSH-Px are data that emphasize the importance of early diagnosis and early treatment in OCD. In addition, the determination of a significant and strong positive correlation between Y-BOCS and HDRS scores and oxidative stress markers IMA and MDA, and a significant and strong negative correlation between them and antioxidants SOD, CAT and GSH-Px, can be interpreted as an indication that oxidative stress increases in proportion to the severity of the disease.

In the treatment of OCD with selective serotonin reuptake inhibitors (SSRIs), only half of the patients respond adequately to treatment (50). This result tells us that the pathophysiology of OCD is much more complex than a simple dysregulation of the serotonergic system. Therefore, dysregulation in the corticostriatal-thalamic circuit consisting of glutamatergic, GABAergic and dopaminergic neurons has been included in the neurobiological model involved in the development of OCD (51). N-acetyl-cysteine (NAC) may have a therapeutic effect in OCD due to its effect on the glutamatergic system and reduction of oxidative stress. Although the results are not conclusive, NAC can be used as a treatment option in OCD today (52). However, fluoxetine, an SSRI, was also found to be effective in reducing oxidative stress and decreasing the severity of OCD symptoms, and improvement was associated with decreased oxidative stress (53). These data not only emphasize the role of oxidative stress in the pathophysiology of OCD, but also point to the importance of evaluating antioxidant agents in the treatment of OCD.

One of the limitations of this study is the low number of participants in the patient population to form the OCD+MDD and OCD-MDD groups. Another limitation is the difficulty in standardizing patients’ lifestyles, diets, and smoking status, which have the potential to affect oxidative stress parameters. In addition, the cross-sectional design of our study is another limitation. It remains unclear whether oxidative stress markers will change or remain the same in longitudinal studies in these patient groups.

In conclusion, future research should focus on recording changes in oxidative parameters during the course of the disease, so that dysfunction related to the oxidative system can be defined as a characteristic feature of the disease. In addition, oxidative parameters should be compared between treated and untreated patients, oxidative parameters should be examined in the presence of comorbidity and variations between OCD subtypes should be determined. It should be remembered that patients’ lifestyles, diets, smoking status, and pre-disease antioxidant levels can all affect the results. We think that the use of antioxidants in addition to treatment may make a positive contribution to the disease process when disorders associated with oxidative stress are detected in patients with OCD. Further prospective studies with larger patient groups and research evaluating drugs with antidepressant and antioxidant effects capable of reducing oxidative stress are now needed to confirm our results and to shed light on the underlying mechanisms.

## Data Availability

The raw data supporting the conclusions of this article will be made available by the authors, without undue reservation.
